# Effects of sensorimotor–cognitive training on balance and gait in patients with idiopathic Parkinson’s disease: a randomized controlled trial

**DOI:** 10.1007/s11845-026-04278-2

**Published:** 2026-03-03

**Authors:** Büşra Seçkinoğulları Korkusuz, Ayla Fil, Süleyman Korkusuz, Ayşenur Özcan, Öznur Yiğit, Gül Yalçın Çakmaklı, Bülent Elibol

**Affiliations:** 1 Kızılcahamam Vocational School of Health Services, Department of Therapy and Rehabilitation, Ankara University, Ankara, Türkiye; 2 Faculty of Physical Therapy and Rehabilitation, Hacettepe University, Ankara, Türkiye; 3 Faculty of Health Sciences, Department of Physiotherapy and Rehabilitation, Atılım University, Ankara, Türkiye; 4 Faculty of Health Sciences, Department of Physiotherapy and Rehabilitation, Çankırı Karatekin University, Çankırı, Türkiye; 5 Faculty of Health Sciences, Department of Audiology, Hacettepe University, Ankara, Türkiye; 6 School of Medicine, Neurology Department, Hacettepe University, Ankara, Türkiye

**Keywords:** Parkinson’s disease, sensorimotor-cognitive training, gait, balance, postural control, exercise

## Abstract

**Background:**

Sensory-motor-cognitive integration deficits are common in Parkinson’s disease(PD) and significantly limit functional performance, highlighting the growing need for holistic training approaches that target these domains simultaneously.

**Aims:**

This study aimed to examine the effects of sensory-motor-cognitive integration training on balance and gait in patients with Parkinson’s disease (PwPD).

**Methods:**

A total of 40 PwPD, aged between 45 and 75 years, were included. The intervention group received 24 sessions (3 days per week for 8 weeks) of 60-minute sensory-motor-cognitive integration training. The control group was placed on a waiting list and continued their routine daily activities. All participants were assessed at baseline, at the end of the 8th week, and at the 12th week. Static posturography was used to evaluate balance [Limits of Stability and the Modified Sensory Integration and Balance Clinical Test] and gait (Walk Across test). Additionally, balance was assessed using the Functional Reach Test (FRT), and gait performance was evaluated with the Modified Dynamic Gait Index (mDGI).

**Results:**

Significant time × group interactions were observed for functional balance and gait outcomes. The intervention group showed significant improvements in FRT and mDGI scores over time (*p* < 0.001), whereas no significant changes were observed in the control group. Static posturography also demonstrated significant time-related changes and group differences for most parameters (*p* < 0.05), except for movement velocity, directional control, and step length symmetry.

**Conclusion:**

These findings suggest that individualized, multidomain sensory–motor–cognitive integration training may represent a particularly effective rehabilitation approach for enhancing balance and gait in PwPD.

## Introductıon

Parkinson’s disease (PD) leads to postural instability due to impaired organization of automatic postural responses, deficits in somatosensory integration and modulation of afferent sensory input, and delayed or diminished postural reactions [1, 2].

As postural instability progresses, patients experience deterioration in several motor functions, most notably in balance and gait, which are essential for independent living. Walking is a complex activity regulated by both subcortical and cortical mechanisms. While subcortical control is automatic, rapid, and relatively resistant to environmental stressors, cortical (i.e., cognitive) control is slower, more effortful, and highly susceptible to interference such as distraction or cognitive load [3, 4].

In addition to cognitive impairments, motor and sensory deficits are core features of PD and play a central role in the emergence of postural instability and gait disturbances. Motor symptoms such as bradykinesia, rigidity, and impaired anticipatory postural adjustments limit patients’ ability to respond effectively to balance threats and dynamic environmental demands. Moreover, sensory impairments, including reduced proprioceptive acuity and defective integration of visual, vestibular, and somatosensory information, compromise postural orientation and feedback-based balance corrections [5, 6]. These impairments can diminish the accuracy of internal body representations and delay motor reactions to postural perturbations, ultimately increasing fall risk and reducing mobility.

In PD, degeneration of basal ganglia circuits compromises automatic gait control, forcing individuals to compensate by relying more heavily on cognitively mediated pathways. This shift increases vulnerability to gait disturbances, especially under cognitively demanding dual-task conditions [7, 8].

Cognitive dysfunction is another prominent and debilitating aspect of PD. Although typically associated with advanced stages, up to 40% of PwPD may experience mild cognitive impairment even in early phases [9]. Furthermore, executive dysfunction, particularly involving working memory, task switching, and attentional control, has been reported even in newly diagnosed patients, reflecting early dopaminergic disruption in fronto-subcortical networks [10, 11].

Physiotherapy and rehabilitation are known to improve gait and balance in PwPD. However, despite the frequent co-occurrence of sensory, motor, and cognitive impairments, physiotherapy approaches that simultaneously address all three domains remain rare. Evidence from other clinical populations supports the effectiveness of multimodal interventions combining sensory, motor, and cognitive components, showing improvements in functional mobility, postural control, and fall reduction [12, 13].

In PwPD, sensory-motor training has been shown to enhance both upper and lower extremity function [14], while cognitive training is associated with improvements in gait and balance performance [15]. These findings highlight that gait is not solely a motor activity, but rather an outcome of dynamic integration among sensory input, motor execution, and cognitive regulation.

It is well established that gait impairments may arise even in the early stages of PD [16]. Although interventions addressing either sensory-motor integration or cognitive dual-task performance individually have been explored, studies that combine all three domains within a single therapeutic framework remain scarce—despite growing interest in such integrative approaches to promote neuroplasticity and functional recovery [17].

In light of the interrelated sensory, motor, and cognitive challenges in PD, the present study aimed to examine the effects of a comprehensive training program that integrates sensory-motor exercises with cognitive tasks on balance and gait performance in PwPD. By simultaneously targeting impairments across these three domains, the intervention was designed to address the complex interplay underlying postural instability and gait disturbances in this population. This study hypothesized that sensorimotor-cognitive integration training would have a significant effect on balance and gait outcomes over time compared to a control group.

## Methods

### Study design and procedure

This single-blind randomized controlled trial included 40 patients aged 45–75 years who were being followed up for PD in the outpatient clinic of the Hacettepe University Department of Neurology. The study included individuals diagnosed with Parkinson’s disease according to the UK Parkinson’s Disease Association Brain Bank diagnostic criteria by a neurologist (GYÇ) specializing in movement disorders. The study was conducted between May 2024 and October 2024. Ethical approval was obtained from the Hacettepe University Clinical Research Ethics Committee, and the study was registered at ClinicalTrials.gov (registration number: NCT06390163).

All participants provided written informed consent before enrollment. Eligible individuals were randomly assigned to either the study or control group by AFB using a random number table. Outcome assessments were performed by SK, who was blinded to group allocation. Treatment programs were administered by BSK, who has seven years of experience in sensory-motor-cognitive integration training. To minimize bias, BSK was not informed of the evaluation results until the study was completed.

**Fig. 1:**
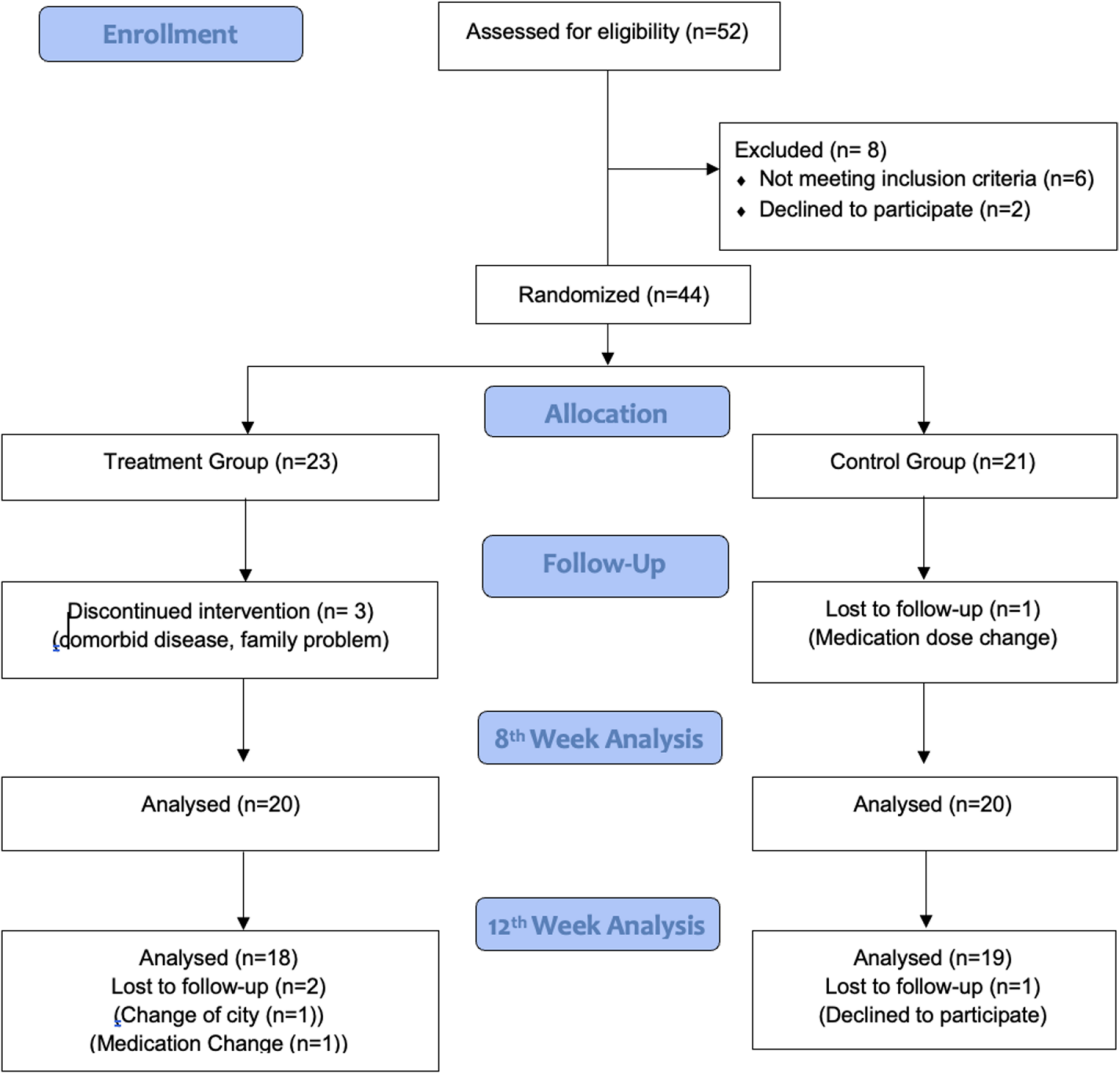
Flow Diagram

A total of 52 patients were assessed for eligibility. Of the 44 patients who met the inclusion criteria and were enrolled, 4 withdrew from the study. Patients were randomized into two groups. Thus, this study included 20 patients in the control group and 20 patients in the treatment group. To distinguish training effects from time-related changes, the control group continued their daily routines without additional intervention and returned for follow-up assessments at weeks 8 and 12. In addition to their usual activities, the study group received sensory–motor–cognitive integration training for 60 min, three times per week, over eight weeks. No additional recommendations were provided to either group. All participants were evaluated at baseline, week 8, and week 12 (Fig. 1).

### Participants

Inclusion criteria were: (1) Modified Hoehn–Yahr Scale (mH&Y) stage between II and III; (2) age between 45 and 75 years; (3) a score of at least 21 on the Montreal Cognitive Assessment Scale; (4) literacy in Turkish; and (5) no change in medication type or dosage during the study period [18, 19].

Exclusion criteria were: (1) presence of other neurological diseases; (2) uncorrected visual problems or vestibular disorders unrelated to PD that could affect balance; (3) serious comorbidities affecting balance and gait or posing life-threatening risks that cannot be controlled with medication (e.g., diabetes, hypertension, cardiopulmonary diseases); (4) long-term corticosteroid use; and (5) orthopedic or systemic diseases that had worsened within the previous six months or caused functional limitations identified on clinical examination.

All participants continued their routine pharmacological treatment during the intervention period. Antiparkinsonian medications included levodopa preparations, dopamine agonists, monoamine oxidase-B inhibitors, and catechol-O-methyltransferase inhibitors, prescribed by their treating neurologists. No changes in medication type or dosage were allowed throughout the intervention period. To ensure comparability between groups, the total daily antiparkinsonian medication dose was calculated as the levodopa equivalent daily dose (LEDD) for each participant. Baseline LEDD values were statistically compared between the intervention and control groups and were found to be comparable.

### Sample size

The required sample size was calculated using G*Power 3.1. Since no studies with identical protocols were found in the literature, a pilot study was conducted with five participants per group to determine the effect size. Data from individuals included in the pilot study were not used in the final analysis. Based on the Limits of Stability – Maximum Excursion (LOS-MXE) values, defined as the primary balance outcome, the effect size was calculated as 1.043. A minimum of 17 participants per group was required to achieve 80% statistical power. To account for possible dropouts, 20 participants were included in each group.

### Intervention procedures and outcome measures

Participants were randomly assigned to either an intervention or a control group. The intervention group received sensory–motor–cognitive integration training for 60 min, three times a week, over 8 weeks (24 sessions), in addition to their usual daily activities. The control group was placed on a waiting list and continued their routine daily activities without additional intervention, returning for follow-up assessments to control for time-related changes. No additional recommendations were provided to either group.

All participants were evaluated at baseline, at the end of week 8, and at week 12. Following the recording demographic and clinical characteristics, balance and gait were assessed using static posturography, including the Limits of Stability (LOS), the Modified Sensory Integration and Balance Clinical Test (mCTSIB), and the Walk Across test. Functional balance was additionally evaluated using the Functional Reach Test (FRT), and gait performance was assessed with the Modified Dynamic Gait Index (mDGI). All assessments and training sessions were conducted during the participants’ “ON” medication phase, approximately one hour after medication intake. Before each assessment, standardized instructions were provided, and no feedback was given to prevent potential learning effects across assessments. The same assessment order and instruments were used at each time point, with rest breaks provided as needed.

#### Sensory-motor-cognitive integration training

The training content was designed by AFB and BSK, who have extensive experience in the PD clinic and in sensory–motor–cognitive integration training. Programs were tailored to each patient’s clinical status. Sessions began with relatively easy tasks that patients could complete successfully, enhancing motivation, and task difficulty was progressively increased throughout the session.

Performance was evaluated based on:

- Time taken to complete the tasks,
- Occurrence of balance disturbances during the tasks,
- Ability to perceive and execute cognitive tasks.

**Fig. 2:**
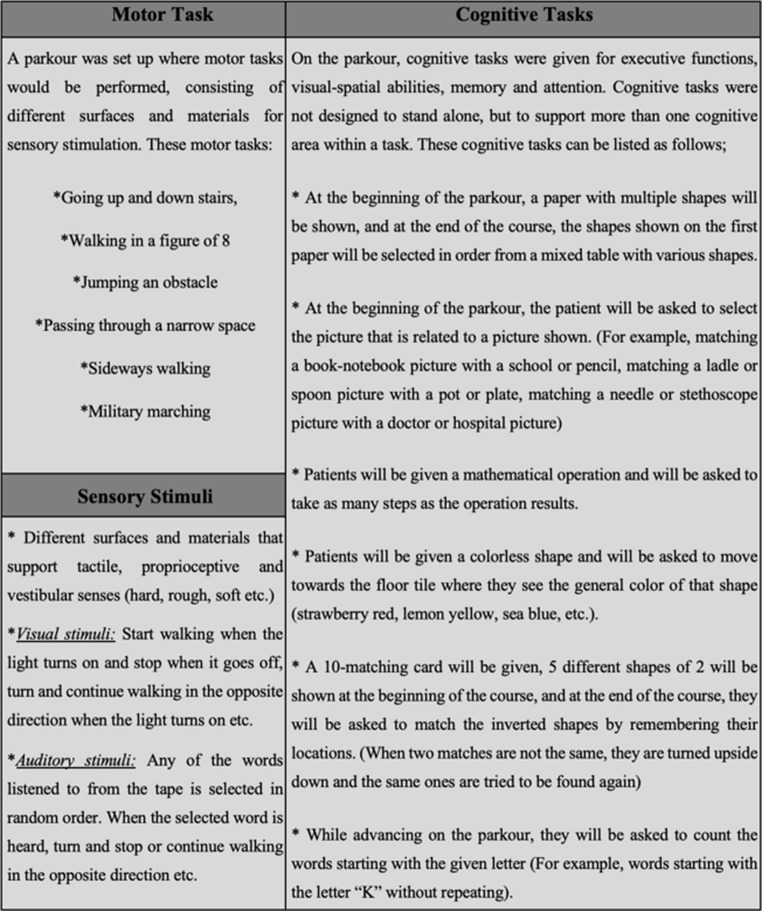
Sensory-motor-cognitive integration training tasks

Each session consisted of an exercise sequence incorporating motor tasks, sensory stimuli, and cognitive challenges. A minimum of three motor tasks, three sensory stimuli, and three cognitive tasks were included in each session, selected according to the patient’s capabilities. Examples of these tasks are presented in Fig. 2.

#### Movement disorders society - unified Parkinson disease rating scale - Part III (MDS-UPDRS-Motor Examination)

This is the revised form of the UPDRS developed in 1980 by the Movement Disorders Society in 2008. The number of questions in the UPDRS, which was 42, was increased to 50 in the MDS-UPDRS, and the yes/no questions were removed because they caused structural inconsistency and were scored between 0 and 4 for all questions. Only Part III, which includes the Motor Examination, was used to assess motor symptom severity, including bradykinesia, rigidity, tremor, posture, gait, and postural stability [20].

#### Static posturography (NeuroCom^®^ Balance Master^®^ Systems)

The Modified Clinical Test of Sensory Interaction on Balance (mCTSIB) and the Limits of Stability (LOS) Test, which are included in the posturography device, were used to quantitatively evaluate static and dynamic balance. Gait was assessed with the Walk Across (WA) test in static posturography.

- *Modified Sensory Integration and Clinical Assessment of Balance Test (mCTSIB)*: In this test, which allows the evaluation of postural oscillations during static posture, each test condition was performed for 10 s with 3 repetitions. The mean postural sway in each condition was calculated in degrees/second (°/sec), and a composite score was also recorded. Evaluation was carried out on the platform on hard and soft surfaces under four different conditions, eyes open and closed [21].
- *Limits of Stability Test (LOS)*: The evaluation was made in 8 directions positioned at 45° angles. The patient was asked to stand still by bringing their heels to the designated squares on the platform and to keep the image representing the center of gravity on the central target. The patient was told that, together with the starting sound of the system, they should move the image on the screen towards the desired target in the fastest and most linear way by shifting the center of gravity and keeping it fixed for 8 s at the point reached. After the evaluations, reaction time (RT), movement velocity (MVL), endpoint excursion (EPE), maximum excursion (MXE), and directional control (DCL) data were obtained [21].
- *Walk Across (WA)*: It was used to evaluate the gait of the patients. During the test, the patient was asked to walk on the platform at their usual walking speed in daily life and then step off the platform. Step width, step length, gait speed, and step length symmetry data were obtained from the evaluation [22].

#### Modified dynamic gait ındex (mDGI)

The Dynamic Gait Index (DGI) is used to assess and document the ability to respond to changing task demands during walking. Although the DGI can predict gait disturbances and the likelihood of falls, a modified version of the DGI (mDGI) was developed due to limitations in the scoring system in the original version of the test. The time, gait pattern (GP), and level of assistance (LOA) for each of the eight DGI tasks have been redefined in the mDGI. The total mDGI score ranges from 0 to 64 points, comprising subscores for time (0–24), gait pattern (0–24), and level of assistance (0–16), with higher scores indicating better gait performance [23].

#### Functional reach test

It is used to evaluate dynamic balance and anteroposterior stability. Patients were asked to raise their arms 90° and extend forward as much as possible while standing, without disturbing the contact of their feet with the ground. Initially, the projection of the acromion and 3rd metacarpal bone on the wall was marked. At the final point after extending forward, the projection of the 3rd metacarpal bone on the wall was marked and the difference between them was calculated. If the reach distance is less than 25.4 cm in elderly individuals, there is a risk of falling, and if it is less than 15 cm, there is a serious risk of falling [24].

### Statistical analysis

Statistical analyses were performed using IBM^®^ SPSS Statistics version 26.0. The normality of data distribution was assessed using the Shapiro–Wilk test and by examining skewness and kurtosis values. Descriptive statistics are presented as mean ± standard deviation (SD) and median (min-max) for numerical data and as frequency (n, %) for categorical variables. Between-group comparisons of demographic and baseline characteristics were conducted using the Fisher exact chi-square test for categorical variables and the Mann–Whitney U test for continuous variables that did not meet the assumption of normality.

A mixed-design repeated-measures analysis of variance (ANOVA) was employed to examine within-group changes over time and group × time interactions for continuous outcome variables. Based on Box’s M test, the assumption of equality of covariance matrices was not violated. Bonferroni adjustment was applied for subsequent multiple comparisons [25]. Statistical significance was set at *p* < 0.05. Partial eta squared (η²p) was reported as the measure of effect size, interpreted as small (0.01 < η²*p* < 0.06), medium (0.06 < η²*p* < 0.14), or large (η²*p* > 0.14) [26].

## Result

The study sample consisted of 21 male and 19 female participants with mH&Y stages 2–3. The two groups were comparable in terms of demographic and clinical characteristics (*p* > 0.05, Table 1).

**Table 1:** Descriptive and baseline clinical characteristics of the control and treatment groups

		Control Group		Treatment Group		pª
		$\:\overline{\mathbf{X}}\:$± SD	𝑋̃ (min–max)	$\:\overline{\mathbf{X}}\:$± SD	𝑋̃ (min–max)	
Age (years)		63.55 ± 8.06	64.5 (45–74)	62.45 ± 9.11	64 (46–74)	0.738
Duration of disease (years)		6.30 ± 3.84	6.5 (1–16)	5.60 ± 3.89	4.5 (1–15)	0.445
LEDD (mg)		947.0 ± 388.12	960 (280–1540)	1003.0 ± 311.44	1035 (430–1460)	0.626
BMI		26.04 ± 2.06	26.5 (20.62–28.30)	25.74 ± 2.24	26.54 (20.62–28.91)	0.121
MoCA		24.85 ± 1.22	25 (22–27)	24.30 ± 1.62	24 (22–29)	0.758
		n	%	n	%	p^b^
Gender	Female Male	8 12	40.0 60.0	11 9	55.0 45.0	0.342
mH&Y	Stage 2 Stage 2.5 Stage 3	4 10 6	20.0 50.0 30.0	5 9 6	25.0 45.0 30.0	0.921
Level of Education	Primary Education High School High Education	6 5 9	30.0 25.0 45.0	7 6 7	35.0 30.0 35.0	0.811
Smoking	No Yes	19 1	95.0 5.0	18 2	90.0 10.0	1.000
Dominant Extremity	Right Left	20 0	100.0 0.0	20 0	100.0 0.0	1.000
Disease Onset	Right Left	11 9	55.0 45.0	10 10	50.0 50.0	0.752

$\:\overline{\mathrm{X}}$± SD: mean ± standard deviation, 𝑋̃(min-max): median(minimum-maximum), n: Number of Patients, LEDD: Levodopa Equivalent Daily Dose, BMI: Body Mass Index, MoCA: Montreal Cognitive Assessment, mH&Y: Modified Hoehn-Yahr Scale, a: Mann-Whitney U Test, b: Fisher exact chi-square test

A significant main effect of time was found for all static posturography parameters, except for the MVL subparameter of LOS. Significant time × group interactions were observed for all static posturography parameters, except for the DCL subparameter of LOS and the step length symmetry subparameter of the WA test (Table 2).

**Table 2:** Comparison of static posturography evaluation results of groups

		Control Group			Treatment Group			Mixed Design Repeated Measures ANOVA					
		$\:\overline{\mathbf{X}}\:$± SD			$\:\overline{\mathbf{X}}\:$± SD			Time Effect			Time x Group Interaction		
		Week 0	Week 8	Week 12	Pre-Treatment	Post-Treatment	Treatment Follow-up	F	p	(ŋ^2^p)	F	p	(ŋ^2^p)
LOS	RT (sec)	1.08 ± 0.33	1.17 ± 0.40	1.19 ± 0.40	1.34 ± 0.53	1.02 ± 0.31	1.02 ± 0.26	3.29	0.043*	0.09	12.89	< 0.001*	0.27
	MVL (deg/sec)	2.45 ± 1.30	2.23 ± 0.85	2.12 ± 0.73	2.24 ± 1.06	3.00 ± 1.03	2.56 ± 0.81	2.16	0.136	0.06	9.49	0.001*	0.21
	EPE (%)	54.40 ± 16.38	56.00 ± 10.58	55.00 ± 11.88	54.80 ± 13.20	68.25 ± 8.46	66.33 ± 9.13	8.94	0.001*	0.20	6.70	0.004*	0.16
	MXE (%)	73.15 ± 14.83	71.50 ± 8.64	73.93 ± 9.32	69.25 ± 13.92	83.45 ± 8.18	84.00 ± 6.65	13.75	0.001*	0.28	22.18	< 0.001*	0.39
	DCL (%)	65.80 ± 13.94	68.05 ± 14.56	70.43 ± 13.89	70.10 ± 12.29	78.90 ± 6.98	78.72 ± 6.04	8.19	0.003*	0.19	2.78	0.090	0.07
m-CTSIB	HG – EO (deg/sec)	0.57 ± 0.66	0.58 ± 0.66	0.61 ± 0.63	0.66 ± 0.58	0.27 ± 0.17	0.22 ± 0.11	8.24	0.004*	0.19	8.21	0.004*	0.19
	HG – EC (deg/sec)	0.53 ± 0.46	0.54 ± 0.39	0.55 ± 0.37	0.58 ± 0.60	0.26 ± 0.16	0.21 ± 0.11	3.81	0.027*	0.10	3.18	0.048*	0.08
	SG – EO (deg/sec)	1.19 ± 0.85	1.18 ± 0.77	1.16 ± 0.73	2.03 ± 1.77	0.68 ± 0.28	0.62 ± 0.20	7.08	0.010*	0.17	7.56	0.008*	0.18
	SG – EC (deg/sec)	2.56 ± 1.53	2.29 ± 1.41	2.06 ± 1.13	3.62 ± 1.86	1.35 ± 0.65	1.16 ± 0.59	27.52	< 0.001*	0.44	15.45	< 0.001*	0.31
	Composite (deg/sec)	1.22 ± 0.70	1.18 ± 0.68	1.11 ± 0.55	1.72 ± 1.04	0.64 ± 0.25	0.57 ± 0.19	18.27	< 0.001*	0.34	14.32	< 0.001*	0.29
WA	Step Width (cm)	19.75 ± 2.44	20.04 ± 3.04	20.14 ± 3.10	20.28 ± 3.09	16.73 ± 3.27	17.67 ± 2.79	5.54	0.009*	0.14	8.12	0.001*	0.19
	Step Length (cm)	40.17 ± 12.61	38.91 ± 10.85	40.33 ± 10.83	37.56 ± 12.43	47.54 ± 12.15	53.16 ± 14.85	13.10	< 0.001*	0.27	15.66	< 0.001*	0.31
	Speed (cm/sec)	52.07 ± 15.19	51.18 ± 12.59	51.02 ± 14.60	48.99 ± 16.32	62.04 ± 16.53	67.36 ± 20.20	9.87	< 0.001*	0.22	11.81	< 0.001*	0.25
	Step Length Symmetry (%)	16.85 ± 11.17	14.75 ± 7.59	16.00 ± 6.62	17.90 ± 9.14	9.80 ± 5.73	9.61 ± 6.35	6.52	0.005*	0.16	2.54	0.099	0.07

$\:\overline{\mathrm{X}}$± SD: mean ± standard deviation, LOS: Limit of Stability, RT: Reaction Time, MVL: Movement Velocity, EPE: Endpoint Excursion, MXE: Maximum Excursion, DCL: Directional Control, m-CTSIB: Modified Clinical Test of Sensory Interaction and Balance, HG: Hard Ground, SG: Soft Ground, EO: Eyes Open, EC: Eyes Close, WA: Walk Across, sec: second, deg: degree, %: percentage, cm: centimeter, ŋ^2^p: Effect size, *:*p* < 0.05

**Fig. 3:**
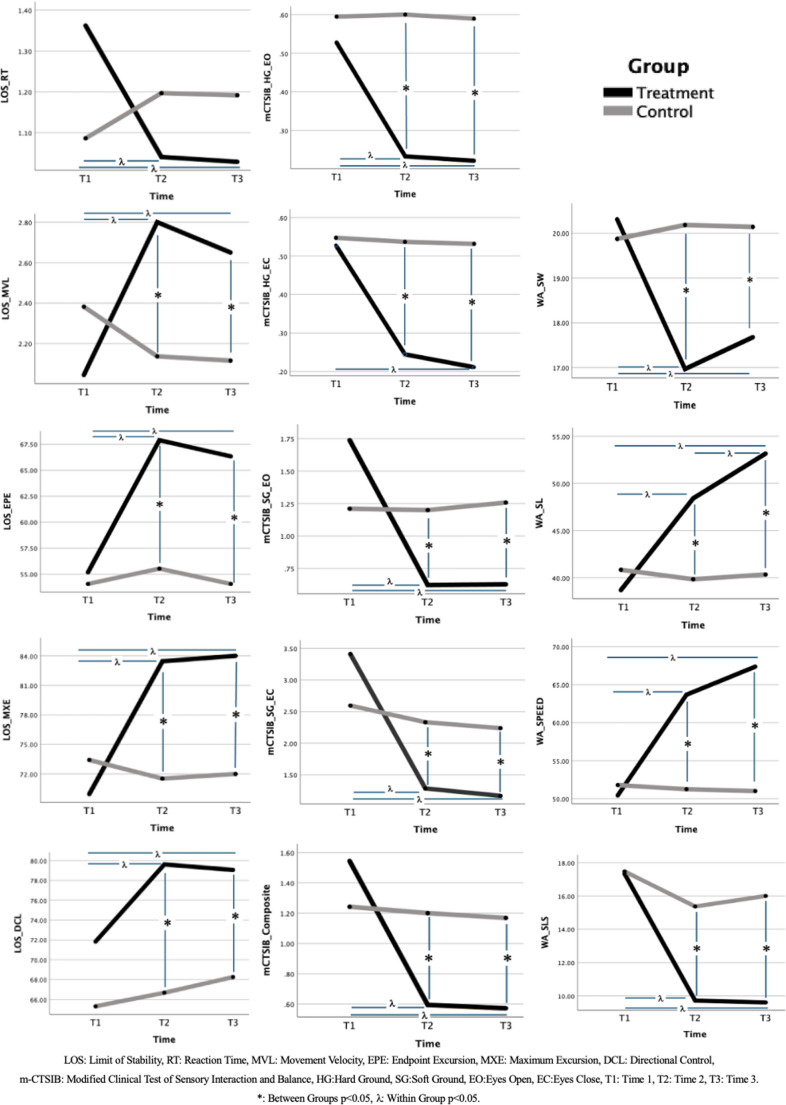
Plots of post-hoc (Adjustment for multiple comparisons: Bonferonni)

Post hoc results for static posturography parameters are illustrated in Fig. 3.

Repeated-measures ANOVA revealed significant main effects of time and significant time × group interactions for MDS-UPDRS, FRT, mDGI-time, mDGI-GP, and mDGI-Total scores (Table 3). While no significant changes were observed over time in the control group, all clinical outcomes improved significantly over time in the treatment group. Post hoc analyses showed that, compared to baseline, the treatment group demonstrated significant improvements in MDS-UPDRS, FRT, mDGI-time, mDGI-GP, and mDGI-Total scores at both post-test (*p* < 0.001, all) and follow-up (*p* < 0.001, all). In the control group, all variables remained statistically unchanged across all time points (*p* > 0.05, all). Although there were no baseline differences between groups (*p* > 0.05, all), the treatment group outperformed the control group at both the second (*p* < 0.05, all) and third evaluations (*p* < 0.05, all).

**Table 3:** Comparison of clinical evaluation results of the groups

	Control Group			Treatment Group			Mixed Design Repeated Measures ANOVA					
	$\:\overline{\mathbf{X}}$± SD			$\:\overline{\mathbf{X}}$± SD			Time Effect			Time x Group Interaction		
Week 0	Week 8	Week 12	Pre-Treatment	Post-Treatment	Treatment Follow-up	F	*p*	(ŋ^2^*p*)		F	*p*	(ŋ^2^*p*)
MDS-UPDRS (Motor)	32.10 ± 10.03	31.25 ± 9.99	31.68 ± 10.44	34.90 ± 10.16	22.05 ± 8.21	22.05 ± 8.85	91.17	< 0.001*	0.72	70.63	< 0.001*	0.67
FRT (cm)	21.17 ± 2.88	21.62 ± 2.38	21.28 ± 2.20	20.35 ± 2.30	24.60 ± 2.74	24.72 ± 3.08	55.39	< 0.001*	0.61	37.33	< 0.001*	0.52
mDGI Time score	12.85 ± 3.92	13.40 ± 3.34	13.21 ± 3.68	11.75 ± 3.59	17.25 ± 3.62	17.83 ± 3.32	89.85	< 0.001*	0.72	60.81	< 0.001*	0.64
mDGI GP score	16.50 ± 3.22	16.80 ± 2.41	16.31 ± 2.63	15.35 ± 3.37	19.55 ± 2.48	19.61 ± 2.78	28.24	< 0.001*	0.45	24.71	< 0.001*	0.41
mDGI LOA score	15.75 ± 0.63	15.90 ± 0.44	15.89 ± 0.46	15.60 ± 1.18	15.95 ± 0.22	16.00 ± 0.00	3.93	0.055	0.10	0.82	0.373	0.02
mDGI Total score	45.10 ± 7.27	46.10 ± 5.59	45.42 ± 6.11	42.70 ± 7.47	52.75 ± 5.78	53.44 ± 5.75	65.80	< 0.001*	0.65	47.23	< 0.001*	0.57

$\:\overline{\mathrm{X}}$± SD: mean ± standard deviation, MDS-UPDRS: Movement Disorder Society -Unified Parkinson’s Disease Rating Scale, FRT: Functional Reach Test, mDGI: Modified Dynamic Gait Index, GP score: gait pattern score; LOA score: level of assistance score, cm: centimeter, ŋ^2^p: Effect size, *:*p* < 0.05

## Discussion

This study provides novel insights into the rehabilitation of patients with Parkinson’s disease (PwPD) by demonstrating that sensory–motor-cognitive integration training can yield significant and lasting improvements in both gait and balance. While numerous interventions have been proposed for PwPD, most focus either on motor rehabilitation or cognitive training in isolation. In contrast, the present study adopts a multidomain approach that simultaneously engages sensory, motor, and cognitive systems—reflecting the complex, multisystem nature of Parkinson’s disease. The integration of cognitive demands into sensorimotor exercises may not only enhance neuroplasticity through dual task learning but also address the real-world challenges PwPD face, where mobility is rarely performed without concurrent cognitive load. To our knowledge, this is the first randomized controlled trial to specifically evaluate the combined effects of sensory–motor and cognitive training on objective posturography measures, dynamic gait index, and functional reach outcomes in this population, revealing both immediate post-intervention gains and short-term retention. These findings support a paradigm shift towards tailored, multifaceted rehabilitation strategies that target the interplay between motor and cognitive domains in PD.

In PD, no single treatment may be appropriate for all patients, and individual differences should be considered for the appropriate treatment of each patient. It is also possible that multidomain approaches targeting a combination of cognitive and motor abilities may improve functional outcomes to a greater extent than single approaches. Considering that PD is more than just a movement disorder, motor-cognitive training may be an important option to improve cognitive function and motor symptoms in PwPD [27, 28]. In addition, training with sensory stimuli facilitates learning in patients and improves gait and functional activities [29, 30]. Considering this information, it can be concluded that flexible treatment programs that include multifaceted sensory-cognitive and motor interventions and are tailored to the needs of the patient may be appropriate for PwPD.

Although there are not enough studies on this subject when the literature is examined, Balkan et al. [31] examined the effects of sensorimotor training given to PwPD in addition to the classical physiotherapy program for 6 weeks. The study results showed that PwPD who received sensorimotor integration training improved balance parameters in clinical and laboratory tests, while no improvement was observed in sensory analyses except for the vestibular system score. Although sensory system scores were not examined individually in this study, improvements were observed in all subtests of the mCTSIB test, which assesses sensory integration under different sensory conditions. We believe that the reason for the improvement in all sensory analyses is that the sensory-motor-cognitive integration training used in the study, included more complex tasks and sensory conditions.

In this study, the mCTSIB test was used to evaluate balance in 4 different conditions and it was observed that the swing speed decreased in all conditions after 8 weeks of training in the treatment group. In the literature, no improvement was observed in swing speed in studies where swing was evaluated with different devices after treadmill, agility, and exercise training [32, 33]. In this study, we think that sensory–motor–cognitive integration training reduced swings in all conditions by stimulating the proprioceptors and providing muscle tone regulation.

In the present study, dynamic balance was evaluated using the Limits of Stability (LOS) test. A significant main effect of time was observed for all LOS parameters except the MVL subparameter, and a significant time × group interaction was found for all parameters except DCL. Previous studies investigating balance-oriented training interventions have yielded heterogeneous results [34–36]. Consistent with our findings, improvements in LOS parameters have been reported in studies employing dynamic posturography [34], and game-based balance training incorporating active visual and auditory sensory cues [35]. In contrast, no improvements were observed in LOS outcomes in a study that implemented specific exercise and rotational training [36], suggesting that the inclusion of sensory cues may play a critical role in enhancing balance performance.

While Shih et al. [35] reported no change in movement velocity, Qutubuddin et al. [34] demonstrated results highly comparable to ours; however, in their study, both assessment and intervention were conducted using the same device, which may have influenced the outcomes. In contrast, our study employed different tools for assessment and intervention, reducing the potential for device-specific learning effects. For this reason, our results are more directly comparable to those of Shih et al. We further propose that the increase in MVL values observed in our cohort may be attributable to the cognitive components integrated into the sensorimotor training protocol. Supporting this notion, Pelosin et al. [37] demonstrated that cognitive dual-task training can enhance gait speed in PwPD, reinforcing the potential role of combined motor-cognitive approaches in improving functional mobility.

In this study, FRT was used to assess anteroposterior dynamic balance and fall risk, and the treatment group demonstrated significant improvements that were preserved at follow-up. The FRT is a commonly used measure of dynamic balance and serves as an indirect indicator of limits of stability (LOS) [38]. Therefore, the parallel improvements observed in both FRT and LOS align with expectations. Previous studies have shown that interventions enhancing sensory input—such as multisensory cue-based balance training [39], Tai Chi [40], and game-based interventions [41]—can improve FRT performance, whereas very short-term sensory cueing alone may not produce measurable changes [42]. Our findings are consistent with the existing literature, demonstrating that our multifaceted treatment approach targeting sensory, cognitive, and motor components improves FRT performance by supporting proprioceptive input, postural control, and attention processes.

In this study, the Walk Across Test was used to evaluate gait, and improvements were observed in all subparameters in the treatment group. In the literature, long-term interventions have generally resulted in improvements across all gait parameters—such as step length, step symmetry, and gait speed—or only in gait speed [22, 43]. However, the decrease in step width observed in this study has not been reported in long-term interventions. We believe that this improvement may be attributed to enhanced gait stability following the observed increase in postural control. When the literature is examined, the minimum clinically significant change in gait speed has been reported as 0.05 m/Sects [15, 44]. In this study, sensory–motor–cognitive integration training resulted in an improvement of 15 cm/Sect. (0.15 m/sec), surpassing the minimum clinically significant threshold.

In this study, the mDGI was used for clinical assessment of gait, and significant improvements were observed in the time and gait pattern subscores and total score of the mDGI, consistent with the Walk Across Test findings. Because the study included individuals with mH&Y stages 2–3, most participants did not require assistive devices at baseline, and therefore, maximum scores were observed in the mDGI level of assistance (LOA) subparameter. Therefore, no additional change was observed in this parameter after treatment. Although no studies have evaluated the effectiveness of treatment with the mDGI in PD, interventions such as lumbosacral mobilization [45], dual-task aquatic exercises [46], and virtual reality rehabilitation [47, 48] that increase sensory input have been shown to increase DGI scores, and our results are consistent with this literature. Given the multifaceted nature of our treatment program targeting sensory, motor, and cognitive components, the improvements observed in all gait subparameters are thought to be a result of this holistic approach. In addition, the fact that the improvements achieved were above the minimal clinically important difference reported for the mDGI (6 points for total, 2 points for the duration subscore) supports the clinical impact of the intervention.

In a recent systematic review, Yau et al. [49] reported that virtual reality interventions demonstrated the strongest effects on UPDRS motor scores and balance measures, while proprioceptive-based approaches were particularly effective in improving gait. Overall, the authors concluded that virtual reality and proprioceptive interventions are among the most promising methods for enhancing motor symptoms, balance, and gait in PD. Consistent with these findings, the combination of proprioceptive stimuli and multisensory inputs used in our study likely contributed to the improvements observed. Notably, despite UPDRS being a relatively coarse clinical measure, observing a meaningful improvement further highlights the clinical relevance of our intervention.

The strongest aspect of this study is that it is the first study to examine the effects of sensory-motor-cognitive integration training on balance and gait in PwPD. Other strengths of the study include the follow-up assessment and the presence of a control group to distinguish training effects from time effects, as well as its randomized, single-blind design. The limitations of this study are that the individuals were not grouped by disease stage, and the study design evaluated only the 1-month protection period rather than the long-term effects of treatment.

## Conclusion

In this study, it is thought that by training multifaceted cognitive functions and using motor and cognitive tasks together with sensory stimuli, sensorimotor integration was improved, and thus motor symptoms, balance, and gait were improved. These findings further support a paradigm shift toward tailored, multidomain rehabilitation strategies that explicitly target the dynamic interplay between sensory, motor, and cognitive domains in Parkinson’s disease. The addition of sensory-motor-cognitive integration to physiotherapy programs in the clinic may be beneficial in reducing PD symptoms and may contribute to the maintenance of gains. Future studies that stratify participants by disease stage and investigate longer-term outcomes would provide clearer evidence regarding the sustained effects and clinical applicability of such multidomain rehabilitation strategies on balance and gait in PD.
